# Biomarker discovery in heterogeneous tissue samples -taking the in-silico deconfounding approach

**DOI:** 10.1186/1471-2105-11-27

**Published:** 2010-01-14

**Authors:** Dirk Repsilber, Sabine Kern, Anna Telaar, Gerhard Walzl, Gillian F Black, Joachim Selbig, Shreemanta K Parida, Stefan HE Kaufmann, Marc Jacobsen

**Affiliations:** 1 Department of Genetics and Biometry, Research Institute for the Biology of Farm Animals, Wilhelm-Stahl Allee 2, D 18196 Dummerstorf, Germany; 2 Bioinformatics Chair, Institute for Biochemistry and Biology at the University of Potsdam, Karl-Liebknecht-Str. 24-25, D 14476 Potsdam-Golm, Germany; 3 Molecular Biology and Human Genetics, University of Stellenbosch, Tygerberg, Cape Town 7505, South Africa; 4 Department of Immunology, Max-Planck-Institute for Infection Biology, Charitéplatz 1, D 10117 Berlin, Germany; 5 Department of Immunology, Bernhard-Nocht-Institute for Tropical Medicine, Bernhard-Nocht-Str. 74, D 20359 Hamburg, Germany

## Abstract

**Background:**

For heterogeneous tissues, such as blood, measurements of gene expression are confounded by relative proportions of cell types involved. Conclusions have to rely on estimation of gene expression signals for homogeneous cell populations, e.g. by applying micro-dissection, fluorescence activated cell sorting, or *in-silico *deconfounding. We studied feasibility and validity of a non-negative matrix decomposition algorithm using experimental gene expression data for blood and sorted cells from the same donor samples. Our objective was to optimize the algorithm regarding detection of differentially expressed genes and to enable its use for classification in the difficult scenario of reversely regulated genes. This would be of importance for the identification of candidate biomarkers in heterogeneous tissues.

**Results:**

Experimental data and simulation studies involving noise parameters estimated from these data revealed that for valid detection of differential gene expression, quantile normalization and use of non-log data are optimal. We demonstrate the feasibility of predicting proportions of constituting cell types from gene expression data of single samples, as a prerequisite for a deconfounding-based classification approach.

Classification cross-validation errors with and without using deconfounding results are reported as well as sample-size dependencies. Implementation of the algorithm, simulation and analysis scripts are available.

**Conclusions:**

The deconfounding algorithm without decorrelation using quantile normalization on non-log data is proposed for biomarkers that are difficult to detect, and for cases where confounding by varying proportions of cell types is the suspected reason. In this case, a deconfounding ranking approach can be used as a powerful alternative to, or complement of, other statistical learning approaches to define candidate biomarkers for molecular diagnosis and prediction in biomedicine, in realistically noisy conditions and with moderate sample sizes.

## Background

**Figure 1:**
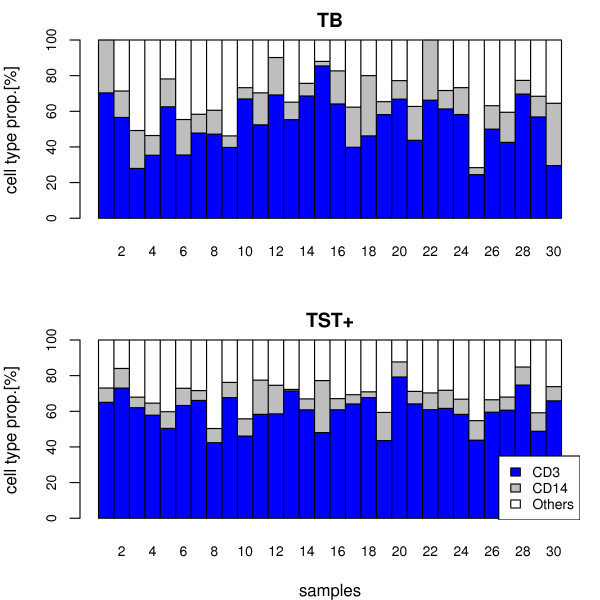
**Experimentally defined proportions of different blood cell types**. Individual proportions of PBMCs are depicted (CD3^+ ^cells, CD14^+ ^cells, and Others) for groups of TB patients and TST+ individuals. Cell type proportions are highly variable, even between individuals within a group.

For studies involving heterogeneous tissue samples, detection of differential gene expression from molecular profiles, as well as identification of biomarkers is a problem of validity: molecular profile variation and changes in cell type proportions between tissue samples are confounded [1-4]. However, heterogeneous tissues are frequently used (e.g. blood, tumor) and further confounded in pathological situations where diseased tissue is frequently infiltrated by immune cell populations. The most widely used material is blood, which is frequently sampled for diagnostic or prognostic purposes. Blood is frequently used as surrogate tissue in many clinical studies for reasons of accessibility, ease of storage and processing. Valid biomarkers from blood are thus often targeted [2]. Regarding tissue heterogeneity, however, blood is an extreme example since inter-individual differences and disease-specific changes, amongst other reasons, lead to high variability in composition ([5,6], cf. our data, figure 1).

**Figure 2:**
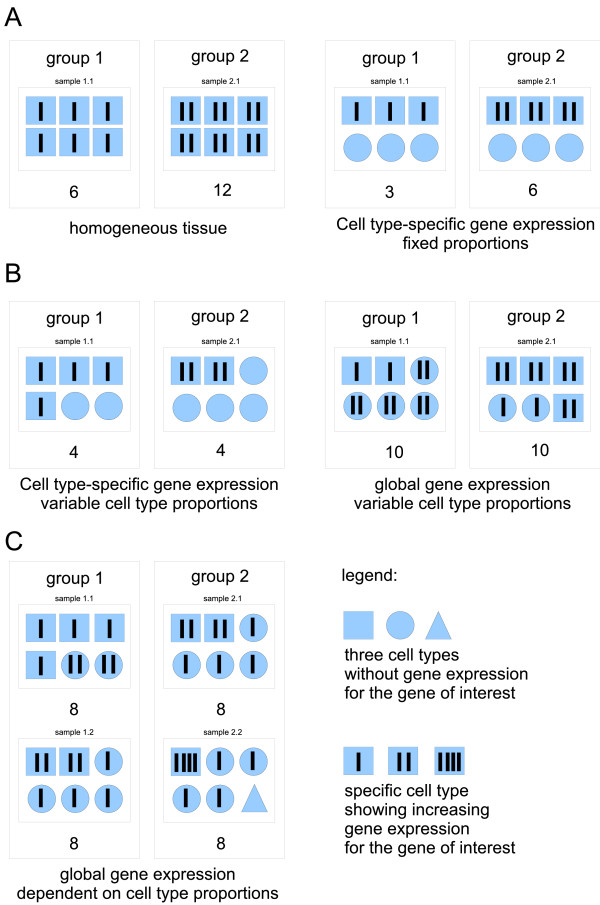
**Cases of gene expression in tissue context**. (A) Non-problematic cases; (B) Confounding of cell type proportions and cell-type specific gene expression: simple and problematic case, deconfounding is possible; (C) worst case: gene expression depends on cell type proportion, deconfounding not possible.

Cell sorting of blood cells, or - in the case of solid tissues - micro-dissection [7], depend on sophisticated equipment. Hence, biomarker studies under field conditions, especially in resource-poor countries, have to rely on molecular profiling from whole blood samples. Ideally, biomarkers with prominent and clear signals can be used which remain detectable in spite of varying cell type populations. However, biomarker signals for more subtle differences are most likely not detectable due to confounding tissue compositions. Figure 2 gives an overview over possible scenarios:

Figure 2A, shows the non-problematic case for homogeneous tissue (e.g. culture of homogeneous cell populations under synchronizing conditions) without any confounding or interpretation problems (left), or tissue with fixed cell type proportions. For these cases, there is no confounding problem [3].

Figure 2B, refers to two cases for which *in-silico *approaches exist for deconfounding: The simple case (figure 2B, left) refers to a situation in which a gene of interest is exclusively expressed in a certain cell type (one amongst others, in varying proportions), and the proportions of this cell type in the study samples have been determined.

Such cell type-specific gene is differentially expressed if the interaction term in the linear model(1)

is significant. Here, *y*_*i *_depicts the log-ratio of gene expression signals for a specific gene in a common reference design (sample *i*), but it could also be a vector of log-intensities for one-color chips after normalization. *β*_0 _is the overall mean for this gene, representing the background signal (without any cells of the cell type exclusively expressing this gene). The binary factor *g *represents patient (*g *= 1) or control status (if *g *= 0) for the respective sample, and *p*_*i *_denotes the proportion of the immune cell population in question as confounding factor. The variable *g *× *p *indicates the interaction effect of study group and immune cell proportion. Finally, *ε*_*i *_denotes the residual for sample *i*. An important assumption for this modeling approach is that single-cell gene expression is independent of cell type proportions. For an example of this type of analysis see the contribution by Jacobsen et al. (2006) [1]. Similar problems and their solutions were presented by Kriete and Boyce (2005) [8] combining tissue composition data and gene expression data, as well as by Gosh (2004) [9], for the latter without estimates of the cell type proportions. If gene expression is no longer restricted to a specific cell type (as in figure 2B, right), we are dealing with the problematic case for which it is harder to disentangle influences of single-cell gene expression and variation in cell type proportions. A few similar approaches exist dealing with such a case, all employing an iterative optimization of the decomposition as given by equation 2:(2)

Here, *X *denotes the classical *gene expression matrix *(genes by samples). *S*, the *signature matrix*, gives the cell type specific gene expression profiles (genes by cell types), and *C*, the *concentration matrix*, gives cell type proportions over samples (cell types by samples).

An alternative formulation is given in equation 3 for the mixture of two cell types (with cell type specific expression signatures *s*_1,*i *_and *s*_2,*i *_for gene *i*):(3)

where  denotes the expression value of the *i*th gene in the *k*th heterogeneous sample and 0 ≤ *c*_*k *_≤ 1 denotes the fraction of the first cell type in the *k*th mixture; equivalent expressions are used in [4,10,11]. Venet et al. (2001) [10] were first to study this approach. In their contribution they made use of a de-correlation approach, which tends to improve the reconstruction of simulated cell type specific gene expression profiles. Experimental data were also used but without the possibility to validate their deconfounding results in a straight forward way. Lu et al. (2003) [11] described a similar approach for analyzing yeast cell cycle expression patterns. Likewise, Stuart et al. [12] investigated prostate tumor tissue. Lahdesmaki et al. (2005) [4] for the first time introduced an approach, which also estimated the appropriate numbers of cell types for deconfounding analysis.

However, none of these prior approaches systematically studied:

- reconstruction of cell type specific gene expression profiles validated with experimental data;

- sample size effects;

- realistic simulation parameter settings derived from appropriate experimental data, with noise conditions as in a typical clinical study;

- the power of detection of differential gene expression in comparison with a classical approach;

- how to use a deconfounding approach in a classification task.

These are the core objectives which our study aims to contribute.

The experimental basis includes an experimental gene expression data set of 40 Agilent two-color arrays for two groups of a field study: tuberculosis patients (denoted TB cases) and healthy household contacts with a positive tuberculin skin test (denoted TST+, healthy controls).This dataset is part of the Grand Challenges in Global Health Project: Grant Number 37772, “Biomarkers of protective immunity against Tuberculosis in the context of HIV/AIDS in Africa” (funded by the Bill & Melinda Gates Foundation through the Grand Challenges in Global Health Initiative). From each of the enrolled individuals, RNA was prepared from a whole-blood sample. From the same samples, cells with active gene expression, peripheral blood mononuclear cells (PBMC), were isolated and cell type proportions determined. CD3^+^-cells (T-lymphocytes) were enriched in these samples and collected for RNA preparation (for more details on the experimental dataset see Methods). Resulting data contain proportion and cell type specific gene expression profile for the most prominent RNA containing cell type in blood, as well as the whole blood gene expression signal of the same samples. This design constitutes a valuable validation dataset for testing and further developing an algorithm for deconfounding, as estimated cell type specific gene expression profiles can be compared to those of FACS-sorted cells.

In our contribution, we study applicability and optimization of the deconfounding approach for detection of differential regulation of features in a univariate approach, as well as an approach using deconfounding for the classification task, towards identification of biomarker panels in heterogeneous tissues.

## Methods

### Experimental data

Gene expression data are part of the Grand Challenges in Global Health Project: Grant Number 37772, “Biomarkers of protective immunity against Tuberculosis in the context of HIV/AIDS in Africa” (funded by the Bill & Melinda Gates Foundation through the Grand Challenges in Global Health Initiative; http://www.biomarkers-for-tb.net/. PBMC from 40 TB cases and from 40 healthy household contact controls were extracted and analyzed by flow cytometry for proportions of CD3^+ ^T-lymphocytes and CD14^+^mononuclear phagocytes as described before [1]. All donors gave informed consent. This study was approved by local ethics committees in Stellenbosch (South Africa) (N05/11/187) and Berlin (EA 10 1/176/07, Germany).

Signals of gene expression in whole blood as well as in CD3^+ ^cells, for the Human Whole Genome Oligo 44K Agilent arrays (GE2_44k_1005) were measured according to manufacturer's protocols. The microarray design was an *independent swop design *as recommended by Landgrebe *et al*. [13]: 50% of each group ("TB", "TST+", "TST-") were labelled with Cy3, the other half using Cy5. Pairs for hybridization on an array were chosen to match regarding age and gender. For validation of the deconfounding algorithm we used CD3^+ ^proportions. CD3^+ ^cells sorted by fluorescence-activated cell sorting (FACS) were subjected to RNA extraction and microarray measurements of gene expression following the same procedure as for the whole blood samples. More details about the observational field study as well as the gene expression dataset will be published separately (see http://www.biomarkers-for-tb.net/publications).

Gene expression data were normalized using R-package *limma *[14]: background correction using the method *normexp *[15], lowess normalization was applied for each array (within array normalisation), quantile normalisation on the set of all arrays (between array normalisation) as recommended [16]. As proposed by [17], gene expression intensities for both groups were obtained as in Equations 4 and 5 from re-parameterizing the normalized log-ratios (M) and mean log-intensities (A) resulting from the *limma *analysis.(4)(5)

Summarizing, for each of the 40 TB cases and the 40 healthy household contact controls we were able to analyze gene expression data of whole blood as well as for the sorted CD3^+ ^cells of the same samples together with their FACS-measured cell type proportions for the CD3^+ ^cell population.

### Deconfounding algorithm: implementation and enhancements

The basis of our deconfounding algorithm was implemented as proposed by Venet et al. (2001) [10] and Lahdesmaki et al. (2005) [4] using R [18]:

   input X and n

  normalize columns of X (either centre, or by quantile normalization)

   generate start values for S and C

   apply constraints to S and C (see below)

(*) fix S, calculate C using lsqnonneg-algorithm

   apply constraints for S

   fix C, calculate S using lsqnonneg-algorithm

   apply constraints for C

   if | X - SC | < a or number iterations > b then EXIT and report S and C

   else continue at (*)

where *X *is the gene expression matrix measured from heterogeneous tissue (rows: genes, columns: samples), *S *and *C *as in equation 2, iteration exit criteria were set *a *= 0.1 and *b *= 100. The Least squares non-negative matrix factorization algorithm is implemented as in the MATLAB function lsqnonneg [19]. The constraints are:

1. *S *non-negative and normalized (either centered, or by quantile normalization [16])

2. 0 ≤ *c*_*ij *_≤ 1 for all elements of *C *(cell type *i*, sample *j*)

3. ∑_*i *_*c_ij _*= 1 for all samples *j *(i.e. cell type proportions sum to 100%)

Our implementation is available as an R-package and has additional options for using quantile normalization instead of global normalization proposed previously [10]. Moreover, it is possible to run the deconfounding on log-values of the normalized intensities or on non-log data. Finally, our implementation does not apply the de-correlation proposed by Venet et al. [10].

To assign the right cell type for each of the estimated profiles, our implementation relies on a majority count decision involving the estimated gene expression profiles from *n*_marker _= 9 markers. Five of these markers are considered to be expressed exclusively for a specific cell type (positive marker genes) and the remaining four exclusively *not *in this cell type (negative marker genes). Marker genes were chosen according to a priori molecular immunological knowledge. For our experimental dataset we used CD3D, CD3E, CD3G, CD2 and CD7 as positive markers, and CD19, FCGR1A, CD14 and MARCO as negative markers for the CD3^+ ^T cells.

### Simulated data

Cell type specific gene expression profiles (columns of the signature matrix *S*) were simulated according to a gamma distribution such that expectation value and variance were those of the experimental data (shape *a *= 12.5 and scale *b *= 0.65):(6)

As by Venet et al. [10], biological variance was modeled as multiplicative error term ϵ, technical variance as additive error term ϵ. For our experimental data, variation was found to increase with mean signal intensities. Therefore, we decided to model a constant coefficient of variation instead of standard deviation:(7)

where *η *= 0.17 and ϵ ~ N(0, *χ *· *I*_gene_), using *χ *= 0.1 as estimated from our experimental data.

Gene expression values for negative marker genes had expression *X*_marker, neg _= 6.0, positive marker genes had *X*_marker, pos _= 12.0 in the expressing cell type - as observed for the marker genes in our experimental study. Cell type proportions, *C*_sim_, were drawn from the uniform distribution between cell type specific maximum and minimum values as in our experimental flow cytometry data. The simulated gene expression matrix, *X*_sim_, was calculated from simulated cell type-specific gene expression profiles, *S*_sim_, and simulated cell type proportions, *C*_sim_, corresponding to equation 2:(8)

To investigate the algorithm's capabilities regarding detection of differential expression of single features and for classification, two groups of gene expression profiles were simulated, e.g. corresponding to TB patients and TST+ controls in our experimental data. We simulated *n*_sample _= 100 individuals in each group. For each gene expression profile *n*_genes _∈ {1000, 10000} genes were considered, with *n*_markers _= 10 and *n*_diff _∈ {20, 600} differentially expressed biomarkers.

**Figure 3:**
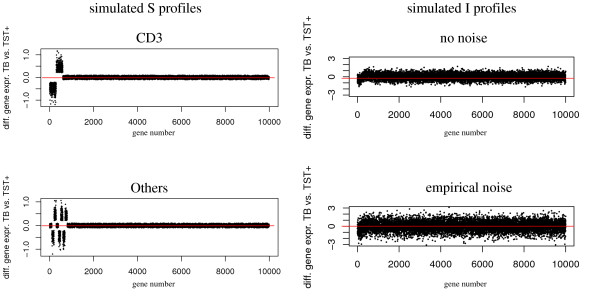
**Simulated cell type-specific gene expression profiles**. Left: *S *matrices for cell-types CD3^+^and Others. Right: *I *matrix before and after adding empirical noise.

Differential expression was simulated by adding Δ_diff _∈ 1, 2, 5} to the expression values of the biomarker genes in the first cell type. Figure 3 illustrates the generation of simulated profiles.

### Power study: valid biomarkers with and without deconfounding

We simulated a gene expression experiment with samples mixed out of two cell types (CD3 and other) for 10,000 genes, where 600 genes were differentially expressed. For the differentially expressed genes we simulated all eight possible combinations of NEUTRAL, UP and DOWN. Sample sizes of the two groups under comparison (alike TB and TST^+ ^healthy control) varied from *n*_samples _∈ {4, 10, 20, 40, 80, 120}. Simulated gene expression data were analyzed as the experimental data. As for the latter we were able to analyze a simulated whole blood sample (mixture of the two cell types) as well as the two cell type-specific gene expression profiles after deconfounding. Simulated whole blood gene expression data were analyzed using the *t*-test, ranking candidates for differential expression using *p*-values and - to enable a direct comparison - considering the 100 top candidates as positive candidates for differential expression. The cell type-specific gene expression profiles (columns of the signature matrix ) estimated from deconfounding were ranked using absolute log-fold-change values. Also here the 100 top candidates were chosen.

### Classification in the case of reversely regulated differentially expressed biomarkers

The worst-case scenario for biomarker detection in heterogeneous tissues arises when cell types involved express differentially regulated biomarkers in opposite directions. In this case, in the tissue RNA isolate, signals for differential expression likely cancel each other and hamper detection of respective biomarkers markedly. To identify a possible exit strategy, we conducted a simulation study for this worst-case scenario, again considering noise values estimated from the experimental data in this study.

To exemplify the worst-case classification task, we simulated differential gene expression as above, but also subtracted the same value from the expression values of the second cell type. This way, for all cells in the mixture averaged over all samples, no differential expression is expected, while for the single cell types it is more or less evident. Gene expression profiles for new samples, for validation of the trained classifiers in the classification scenario, were generated using the identical signature matrices, *S*_sim_, as for the training step, but with new values for the concentration matrices as well as for the noise term realizations.

#### Canonical classification approach

For feature selection, *t*-tests were used to identify biomarker candidates from the simulated heterogeneous tissue gene expression data: The top *n*_cand _ϵ {10, 20} were chosen to train a linear discriminant function as classificator. Classification errors in a validation (500 new cases simulated) for this classical classification approach were then compared to a deconfounding ranking approach, which is described in the following.

#### Deconfounding ranking approach

For the training dataset, a deconfounding analysis was run and *n*_cand _candidates top ranked for differential expression were picked from gene-wise mean absolute differences between the corresponding columns of the estimated signature matrices, , for the two groups. In addition, using the simulated whole blood expression data, *X*_sim_, from the training dataset, a random forest predictor was trained to estimate the cell type proportions , resulting from the deconfounding algorithm run from the same training-data [20,21].

Input to this statistical learning step were the gene expression data in *X*_sim _for the *n*_markers _= 20 marker genes. For each new individual during the validation part of the study, cell type proportions were estimated from the simulated whole blood gene expression profile using the trained random forest machine. Deconfounding results  of the training dataset for the two groups A and B were then multiplied with the estimated cell type proportions for the new individual, to result in group-specific gene expression profiles  and with  with the cell type proportions of the sample in question. The actual gene expression signals of the sample at the chosen *n*_cand _biomarker loci were then compared to these group-specific gene expression matrices and the following summary score computed:(9)

Classification was based on choosing the group for which *γ*_group _was minimal.

### Implementation and availability

R-package deconf implementing the deconfounding algorithm and options, R-scripts for data simulation, data analysis and an anonymized part of the experimental dataset is available as additional file 1 (Windows R-package) and additional file 2 (tar-gz archive).

## Results

As the experimental data offered gene expression profiles for whole blood, i.e. a heterogeneous tissue which is a mixture of several cell types, and in addition the gene expression profiles from CD3^+ ^cells of the same samples, and the respective CD3^+ ^proportions (determined by FACS), we were able to use this information as a basis for a validation study for the proposed deconfounding algorithm.

In addition, to methodologically optimize the deconfounding algorithm as well as to investigate its usability to detect differentially expressed genes and biomarkers usable for classification of new patients (with only whole blood expression profiles measured) - we had to rely on simulation studies.

Summarizing, our study was designed to answer four questions. For which data scale and algorithm settings do we achieve:

- The best estimate of cell type-specific expression profiles (columns of signature matrix)? Data basis: experimental data.

- The best marker-based identification of reconstructed cell type-specific gene expression profiles (columns of )? Data basis: simulation study (parameters estimated from experimental data).

- The largest power to detect differential expression? Data basis: simulation study (parameters estimated from experimental data).

- The smallest prediction errors for the classification task? Data basis: simulation study (parameters estimated from experimental data).

### Reconstruction of cell type-specific gene expression profiles and cell type proportions in experimental data

**Figure 4:**
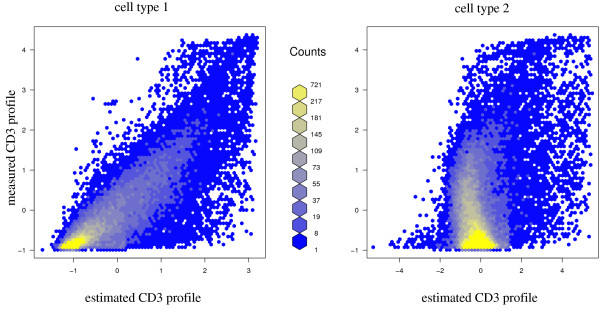
**Validation of estimated gene expression profiles**. Validation of gene expression profile estimates with experimental data from FACS sorted CD3^+^cells: Left panel: measured gene expression intensities for CD3^+^cells versus intensities estimated for cell type 1. Right panel: measured gene expression intensities for CD3^+^cells versus intensities estimated for cell type 2.

**Figure 5:**
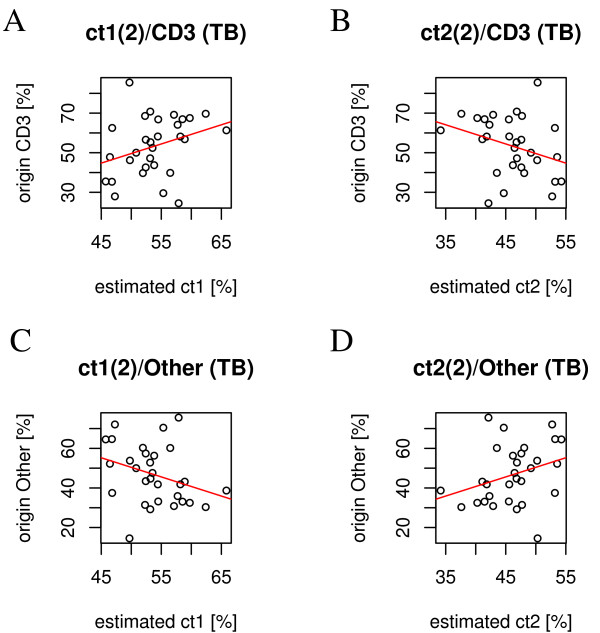
**Validation of estimated cell type proportions**. Validation of cell type proportion estimates with experimental data (FACS counts for CD3^+^cells): A: measured proportion of CD3^+ ^cells versus estimated proportions for cell type 1. B: measured proportion of CD3^+ ^cells versus estimated proportions for cell type 2. C: measured proportion of non-CD3^+^cells versus estimated proportions for cell type 1. D: measured proportion of non-CD3^+^cells versus estimated proportions for cell type 2. Linear regression lines are displayed in red.

The deconfounding algorithm was applied to the whole blood gene expression matrices for both groups of individuals (TB and TST^+^) both using the quantile normalization as well as global mean normalization approach for log- and non-log-intensities. Numbers of cell types was set to *n*_CT _= 2. Deconfounding results - estimated cell type-specific gene expression profiles  as well as cell type proportions  - could be compared to the actual experimental data (see figure 4, figure 5 and table 1):

**Table 1:** Profile reconstruction versus differential gene expression: alternatives for deconfounding algorithm settings

	Optimal deconfounding algorithm settings			
	**log/quant**	**log/not quant**	**not log/quant**	**not log/not quant**

cor reconstr	0.86	0.85	0.73	0.68
DGE power	0.47	0.46	70	0.62

Correlations of measured and estimated cell type-specific gene expression profiles ("cor reconstr.") as well as power for detection of differential expression ("DGE power", see text) -- for all four combinations of using logs or not, applying quantile or global mean normalization, respectively, for the deconfounding algorithm.

Figure 4 displays mean values of the measured CD3^+ ^expression profile in TB patients against both estimated columns of the signature matrix .

For the displayed example in figure 4, non-log data were quantile normalized: Experimental data show considerable variation when compared to the estimates after deconfounding. As expected, cell type 1 is evidently better correlated with the experimental CD3^+ ^profile than cell type 2. The correlation is best for large expression values.

Also, referring to figure 5, even though there is lower correlation between experimental and estimated cell type proportions, the indicated regression lines in the scatter plots for experimental and estimated proportions show the correct tendencies for the respective cell types.

Table 1 (first row) depicts correlations of mean measured profiles with the estimates from deconfounding results for the comparison between the four methodological algorithmic alternatives.

### Deconfounding quality as function of sample size (simulation study)

**Figure 6:**
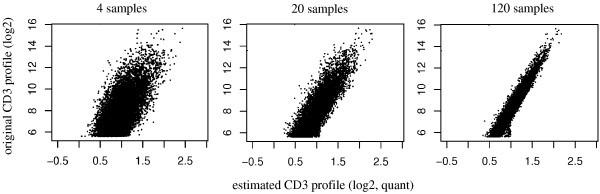
**Profile estimates for simulated data**. Gene expression profile estimates for simulated data, realistic noise, quantile normalisation, and sample sizes of 4 (left panel), 20 (middle panel), or 120 samples (right panel).

To investigate the influence of sample size on the quality of deconfounding results, we had to rely on simulation studies which were aimed at mirroring experimental data distribution and noise as realistically as possible. Figure 6 (middle panel) shows the simulation results for *n*_sample _= 20, which approximates the sample size for the GC6 experimental data (cf. figure 4) - and also a typical value for such type of clinical study involving high-throughput analyses. The effect of sample size is clearly distinguishable for simulation results using *n*_sample _= 4 (figure 6, left) and *n*_sample _= 120 (figure 6, right) respectively.

### Cell type assignment using markers (simulation study)

**Figure 7:**
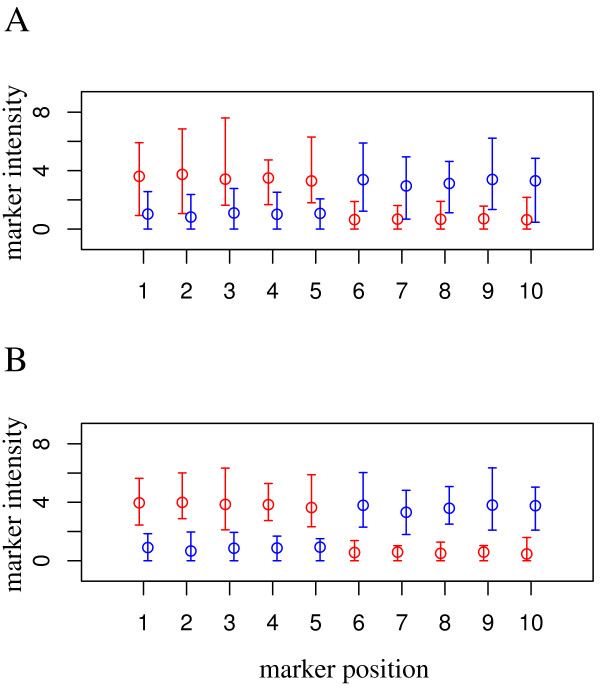
**Cell type-specific assignment using markers**. Mean and range for marker intensities after deconfounding without (A) and with quantile normalization (B). Cell-types CD3^+^(red) and Other (blue). The first five marker positions are positive markers (exclusively expressed in CD3^+^), the remaining five are negative markers (not expressed in CD3^+^).

The deconfounding algorithm itself does not assign a cell type to the estimated cell type specific expression profiles (columns of ). Therefore, to find out in which of the two possible orders the two estimated cell type profiles (CD3^+ ^and others) reside, one has to rely on expression signals of cell type specific markers. Regarding the analysis of the experimental data, such markers were chosen based on *a priori *immunological knowledge. In our simulation studies, we simulated 5-10 positive CD3^+ ^marker genes, which were expressed at high levels (simulated level for *X*_marker, CD3 _= 12), whereas these marker genes showed a low mean expression in the alternative cell type (simulated level for *X*_marker, other _= 6). These expression levels were used as observed for the experimental data. Another group of marker genes was simulated in the reverse manner. Figure 7 shows the distributions of estimated marker gene expression levels from simulated data after deconfounding employing global mean (A) or quantile normalization (B). Here, use of the robust quantile normalization was rewarding for this critical step: Lack of a possibility to assign the right cell types thwarts the analysis as a whole. It is also evident, that marker gene expression levels were estimated mostly correctly regarding relative values in both cell types, whereas absolute gene expression levels were scaled down in the estimates. However, to be able to use these cell type specific estimated marker gene expression levels to assign the right cell types it is only necessary that positive markers have top expression levels in the cell type exclusively expressing them.

### Valid detection of cell type-specific differential gene expression (simulation study)

**Figure 8:**
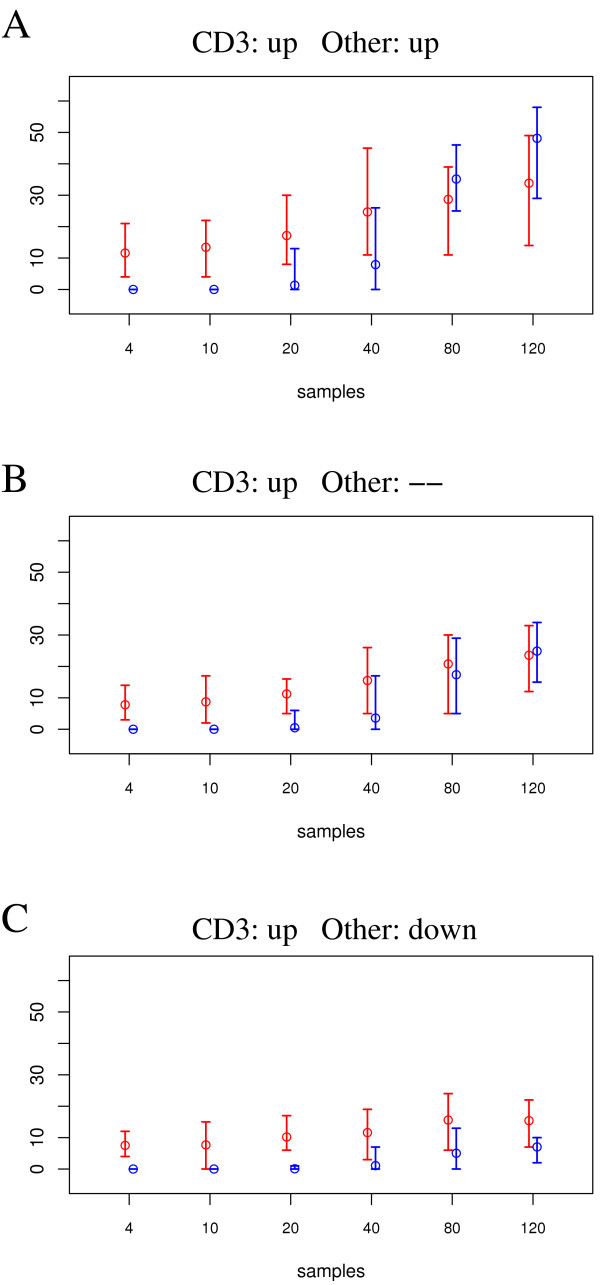
**Power comparison for detection of differential gene expression**. Power comparison for detection of differentially expressed genes in the simulation study with realistic noise. Three cases: (A) gene is up-regulated in both cell-types, CD3^+^and Other; (B) up-regulation only in CD3^+^cell-type, no regulation in Other; (C) up-regulation in CD3^+^, but down-regulation in Other. Mean values and range of numbers of detected candidates are displayed.

Because we want to study the use of deconfounding for biomarker discovery, in our power-study we compared the *t*-test and our deconfounding approach regarding their power to detect differential gene expression (candidate biomarkers). Figure 8 shows the central results: *t*-test and deconfounding approach show comparable results for higher sample sizes (40 ≤ *n*_sample _≤ 120) and cases A and B, for which differential gene expression is either in the same direction in both cell-types or differential in one cell type only. However, for small sample sizes in all cases, and especially also for large sample sizes in figure 8C, the deconfounding ranking approach detects more of the true differentially expressed genes than the *t*-test. As it is for this worst-case scenario (figure 8C), where differentially expressed signals of the cell types involved cancel each other, we aimed at assessing application of the deconfounding ranking approach for the *classification objective *for this case.

As power for detection of differential expression ("DGE power") we define the proportion of truly differentially expressed genes in the 100 top-ranked 100 candidates. Table 1 (second row) depicts this power for detection of differential expression for four algorithmic alternatives. Choosing quantile normalization for intensity values and using non-log values gives optimal results.

### Applying the deconfounding approach for classification (simulation study)

As an important objective is to find biomarkers from the estimated cell type specific gene expression signatures resulting from the deconfounding, we have to show how such biomarkers could be applied to a *new *patient's whole blood expression dataset. The deconfounding algorithm results in estimates for the signature matrix and the concentration matrix for a given group of samples. In our case, the procedure uses simulated gene expression profiles of 40 individuals (per study group) to estimate two cell type-specific gene expression profiles (CD3+ and NotCD3+). It is, however, not possible to use a single individual's profile for deconfounding, as for a single case there is no information available about how a change in cell type proportions influences measured gene expression signals. To enable the use of the deconfounding results for classification of a new individual, we have to either measure or estimate a single individual's cell type proportions. To estimate cell type proportions from a single whole blood expression profile we employed a random forest machine to learn to predict cell type proportions from simulated whole blood gene expression data using the training dataset and the deconfounding estimates of . For a new individual, this trained random forest was then used to estimate cell type proportions.

These were multiplied to the group-specific signature matrices estimated by deconfounding from the two groups in the training data. The resulting group-specific gene expression matrices - based on cell type proportions as in the new individual - were used in a majority votes comparison approach and the individual classified accordingly.

**Figure 9:**
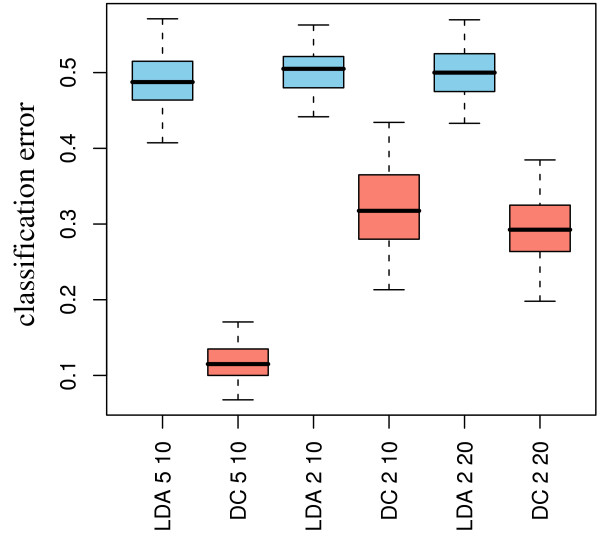
**Applying the deconfounding approach for classification: defining biosignatures in a simulated scenario**. Classification error rates in a simulated scenario using realistic empirical noise and differentially expressed genes, which are reversely regulated in a mixture of two cell-types for a *t*-test-LDA approach (blue boxes) and a deconfounded-biosignature approach (red boxes). Boxplots comprise median, 1^st ^and 3^rd ^quartiles, as well as the 95% confidence interval (assuming normality).

We show that this deconfounding ranking approach significantly improves classification results regarding prediction error rates, if the differential expression of a biomarker panel relies on genes that are regulated in the opposite direction in the cell types involved. Figure 9 shows distributions of classification errors in 100 validation runs. Clearly, the *t*-test-LDA approach is not better than mere guessing, whereas -dependent on noise and numbers of differentially expressed genes - the deconfounding ranking approach correctly classifies most of the simulated cases.

### Predicting cell type proportions in a single whole blood profile in experimental data

**Figure 10:**
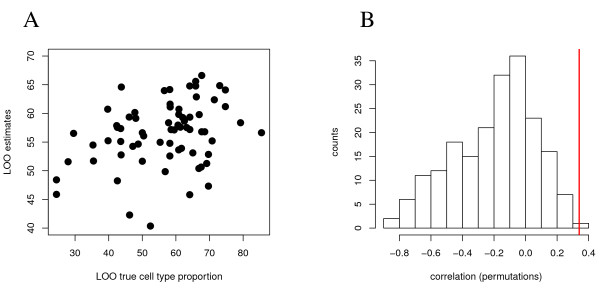
**Prediction of cell type proportions of single whole blood marker expression profiles**. A leave-one-out cross validation approach was used to predict cell type proportions (CD3+) in single samples from their marker gene expression profiles (CD3G and MARCO). A: Scatterplot of estimated CD3+ proportions against true proportions (*r *= 0.34). B: Significance of this prediction precision based on 200 permutations of the true CD3+ proportions and identical analysis as for (A).

We also regressed cell type proportions on marker gene expression (CD3G and MARCO) in the experimental whole blood dataset and achieved a correlation of 34% between leave-one-out samples and their estimated proportions of CD3+ cells. Figure 10 shows a scatterplot of the leave-one-out samples and their estimated proportions, as well as the distribution of correlations with 200 permutated values for the cell type proportions. Prediction is significant, and its precision comparable to what the deconfounding is able to reproduce in the simulated data (compare figure 5).

## Discussion

Gene expression in heterogeneous tissues thwarts valid interpretation of results, detection of differential expression, especially cell type specific regulation in opposite directions, and hence represents a major obstacle towards definition of biomarkers in difficult cases. We propose a modified version of an *in-silico *deconfounding ranking approach which estimates cell type specific gene expression profiles from tissue expression data, even under realistic noisy conditions. We were able to validate these results with experimental data, both from heterogeneous tissue (peripheral blood) and sorted cells. In a realistically simulated example we show how deconfounding ranking can help in detecting differential gene expression in heterogeneous tissues. We developed an approach to use deconfounding results for the task of finding biomarker candidates for classification of a new patient on the basis of his whole blood gene expression profile and information about his cell type proportions (either predicted or measured): This way deconfounding ranking can propose biomarker signatures even in the worst-case scenario where biomarkers are regulated in opposite directions in different tissue cell-types under investigation. The resulting tissue specific biomarkers can be considered as an initial step for the identification of candidate biomarkers for classification. Clearly, any candidate molecular biomarker has to be tested against existing markers, especially clinical markers, and demonstrate a diagnostic or prognostic gain. However, in our contribution we targeted the principal problem of detection of molecular biomarkers from heterogeneous tissue. Our experimental example and the simulation studies demonstrate the problem of confounding cell type proportions and a solution approach using the in-silico deconfounding approach. Our results show that by estimating cell type proportions and cell type specific gene expression patterns, the search for biomarker candidates for classification can be significantly enhanced.

### Significance and applicability of the proposed deconfounding ranking approach

For the purpose of biomarker detection, homogeneous cell populations are not generally a prerequisite as there may be markers so clear that their signal can be read in spite of the considerable variation introduced by tissue heterogeneity. This is mostly a desired result. However, especially in experiments where biomarkers are sought for cases which are not easily separable otherwise (e.g. prospective studies), they might be detected better after taking tissue heterogeneity into account - with our work and manuscript we want to propose an approach for such cases.

Others have implemented and studied principles of *in-silico *deconfounding [4,8-12,22], but our study for the first time combines the following results:

- validates *in-silico *deconfounding results using experimental data of a molecular field study;

- implements a realistic simulation study with noise parameters estimated from the experimental dataset;

- systematically investigates the influence of sample size on quality of estimated cell type specific gene expression profiles;

- compares the power to detect differential expression (i.e. univariate biomarker candidates) with a classical *t*-test approach;

- optimizes the deconfounding algorithm employing a quantile normalization step as well as marker-assisted cell type profile recognition under realistic noise conditions;

- proposes a classification approach using the results of a deconfounding ranking analysis and compares these results with a classical *t*-test-LDA approach for the worst-case scenario of biomarkers regulated in opposite directions.

Our results show that, even under noisy, realistic conditions of a molecular field study - involving field-collected whole blood samples and considerable individual variations between enrolled individuals -the deconfounding ranking approach using non-log, quantile-normalized gene expression data from whole-blood RNA can facilitate identification of valid differential gene expression signals. These biomarker candidates can then be used in a classification approach which - for the case where biomarkers are regulated in opposite directions in different cell-types - is far more powerful than canonical discriminant analysis. In the applied clinical situation, our approach will of course be not more than an initial step for the identification of candidate biomarkers for classification - which then would be entered into further validation studies before applicable for cost efficient clinical routine diagnostics.

### Methodological constraints and requirements

A critical prerequisite of our deconfounding approach is that, in principle, we assume independence of a cell type-specific gene expression profile and the proportion of the respective cell type within the heterogeneous tissue. Figure 2C, illustrates the unfavorable case for which gene expression on the single-cell level is regulated as a function of the expressing cell-type's proportion in the tissue. It is conceivable that such a regulation is indeed real for some genes - and this would not only blur estimates of cell type-specific gene expression profiles, but also produce false estimates for such specific genes. As shown in our validation study, however, in general the independence assumption does not lead to false results for the estimated profile as a whole. Thus, biosignature detection will still be enhanced by use of deconfounding ranking even if the independence assumption for single-cell gene expression and cell type proportion does not hold in every respect.

Some methodological details of our study remain an illustrative approach, and further investigations are thus called for. The normalization procedure has apparent influence on the quality of cell specific profile reconstruction as well as on the power of detection of differential expression. Our decision to use quantile normalization was based on the finding that using the original overall mean normalization by Venet et al. [10] led to poor recognition of cell-types using marker gene expression signals. Single outlier measurements could significantly shift the whole profile, thus thwarting cell-type identification. The quantile normalization approach resulted in a robust, more reliable marker-assisted cell type recognition. An improvement of the algorithm's capability to reconstruct cell type-specific gene expression profiles could be obtained if the starting profiles for the iterative optimization were already seeded with an approximate guess of what the specific cell type profile may look like. Such information could be provided by FACS analysis, or by expression profiles available in the literature (see for example the work of Watkins et al., 2009 [23]). Caution, however, is necessary to avoid inadequate influences on study group-specific differences. Also, averaging multiple deconfounding optimization runs could lead to a stabilizing effect for the resulting predicted cell-type profiles. Here as well, detailed studies are necessary.

Estimates of cell type-specific gene expression profiles were optimal given that deconfounding was run on log-intensities, whereas detection of differential expression was optimal using non-log input values. We may speculate about the reasons for this difference: Possibly, non-log inputs filter out or down-weight small expression values - which in turn often play a minor role in differential expression.

For the simulated worst-case scenarios, i.e. genes which are reciprocally regulated in the participating cell types, the deconfounding ranking approach produced promising results - both for achieving valid estimates of differential gene expression and for the classification task. However, the existing implementation could be improved by implementing a bootstrap test for differential expression, such that not only a ranking of candidates for differential expression, but also an estimate of the number of differentially expressed features becomes feasible. A first approach could be to draw bootstrap samples and compute 95% confidence intervals as quantiles from the bootstrap distribution of the resulting bootstrap estimates for  (*b *denoting a bootstrap index). Such a bootstrap approach could also enable analysis of gene set enrichment with currently available methods (e.g. [24,25]).

### Outlook

The proposed deconfounding ranking approach to classification has to be considered as a first heuristic approach. Its performance sufficiently demonstrates superiority over approaches that do not take into account confounding with cell-type proportions (figure 9). However, a multivariate model of gene expression patterns (biosignatures) is still missing. It would be desirable to arrive at an analysis interface enabling the use of the plethora of available statistical learning methods. Also, the classification approach is dependent on either measurements or estimates of cell type proportions in the sample that is to be classified. If the field of application was gene expression signatures in blood, it is certainly conceivable that a cell type proportions profile is measured, as the necessary laboratory equipment is now available in labs all over the world. However, in our work we propose to try a regression approach based on the expression profiles of the marker genes which are also used to identify the cell type specific expression signatures after deconfounding. This approach worked well for our simulation study, figure 10 shows that it also delivers sufficient results for experimental data - comparable to what the deconfounding algorithm delivers (compare figure 5). However, there is certainly room for improvement - as apparently better estimates of cell type proportions based on single sample whole blood expression profiles would enable improved classification performance.

The presence of up- and down-regulated biomarkers suggest two further possible improvements. First, gene filtering with regard to absolut expression signals, i.e. focussing on medium to highly expressed genes may provide more robust signatures. Second, the identification of gene pairs as in the top scoring pair method [26,27] may be an alternative to the ranking approach taken in our initial study here - and improve reliability in the presence of noisy field measurements.

There also exist alternative approaches to the non-negative matrix factorization approach taken by us and [4,10]. For example, Ghosh proposes a mixture model approach [9], and there also exist Bayesian approaches for this task [22,28]. A comparison of existing methods for the application with biological data from heterogeneous tissues would certainly be an exciting and rewarding field of further work. Especially modern Bayesian methods promise to further improve the results, also regarding more than two cell types in the heterogeneous tissue to be resolved.

In our contribution, the deconfounding ranking approach is applied to gene expression profiles in peripheral blood samples. In principle, it is also applicable for other molecular profiles from heterogeneous tissues, e.g. metabolome or proteome profiles.

## Conclusions

In heterogeneous tissue samples, molecular profiling is confounded by variable cell type proportions. If confounding is severe, as in the important surrogate tissue blood, valid molecular profile measurements are hampered. If micro-dissection or cell sorting are unavailable or too expensive, *in-silico *deconfounding offers an alternative. We have demonstrated possible algorithmic adjustments and approaches for detection of cell type-specific differential gene expression and for molecular profile-based classification. Both these objectives have not been studied previously for approaches of *in-silico *deconfounding. The vigor of our study rests in the use of an experimental validation dataset, which also served to select appropriate realistic simulation parameters to emulate conditions of a molecular field study.

## Authors' contributions

DR conceived the study, ideas of approaches, design and coordination, ran part of the simulation studies and prepared the manuscript. SKe performed most of the statistical programming, implementation of algorithms, estimation of simulation parameters and conduction of simulations. Most of the work in this contribution was part of SKe's diploma thesis at the University of Potsdam. AT summarized the existing R-scripts to form a publishable R-package. GW designed the clinical study and supervised sample preparations in the laboratory, GB managed and supervised the clinical study. MJ contributed significantly to the development of our deconfounding approaches, co-organized the data collection and helped to draft the manuscript. SHEK supervised the Grand Challenges consortium GC6: "Biomarkers for protection against Tuberculosis on the background of AIDS/HIV in Africa" and helped in designing the study and writing the manuscript. SKP significantly contributed with scientific input and design of the GC6 study as well as project coordination. JS contributed to study design and methodological discussions and helped prepare the manuscript. All authors read and approved the final manuscript.

## Supplementary Material

Additional file 1 **R-package ****deconf****(Windows) including example data and script**. *R*-package deconf (Windows version) which implements the deconfounding algorithm together with options for normalization, run-time options for the iteration process, and number of cell-type specific gene expression profiles to be estimated. Also, some toy examples and part of the experimental dataset are included together with executable example scripts for demonstration purposes. Click here for file

Additional file 2 **R-package ****deconf****(tar-gz archive)**. *R*-package deconf (tar-gz archive) Click here for file
