# Supplementary material for: Biomarker discovery in heterogeneous tissue samples -taking the in-silico deconfounding approach
Source: BMC Bioinformatics. 2010 Jan 14;11:27. doi: 10.1186/1471-2105-11-27 (PMC3098067; doi:10.1186/1471-2105-11-27)
Supplement: Additional file 1 — R-package deconf(Windows) including example data and script. R-package deconf (Windows version) which implements the deconfounding algorithm together with options for normalization, run-time options for the iteration process, and number of cell-type specific gene expression profiles to be estimated. Also, some toy examples and part of the experimental dataset are included together with executable example scripts for demonstration purposes. [file 1471-2105-11-27-S1.ZIP › deconf/html/00Index.html]

R: decomposition (deconfounding) of OMICS datasets in heterogeneous
tissues

# decomposition (deconfounding) of OMICS datasets in heterogeneous tissues

---

## Documentation for package ‘deconf’ version 1.0

## Help Pages

|  |  |
| --- | --- |
| deconf-package | package deconf contains function "deconfounding", implementing the decomposition of OMICS datasets of heterogeneous tissues in signature and cell type concentration |
| CELL | Gene expression data for CD3 cells in blood |
| deconf | package deconf contains function "deconfounding", implementing the decomposition of OMICS datasets of heterogeneous tissues in signature and cell type concentration |
| deconfounding | Function decomposing an OMICS dataset from mixed tissue |
| stat | phenotype data for TISS and CELL datasets |
| TISS | Example of experimental gene expression data |
