# Supplementary material for: Biomarker discovery in heterogeneous tissue samples -taking the in-silico deconfounding approach
Source: BMC Bioinformatics. 2010 Jan 14;11:27. doi: 10.1186/1471-2105-11-27 (PMC3098067; doi:10.1186/1471-2105-11-27)
Supplement: Additional file 1 — R-package deconf(Windows) including example data and script. R-package deconf (Windows version) which implements the deconfounding algorithm together with options for normalization, run-time options for the iteration process, and number of cell-type specific gene expression profiles to be estimated. Also, some toy examples and part of the experimental dataset are included together with executable example scripts for demonstration purposes. [file 1471-2105-11-27-S1.ZIP › deconf/html/CELL.html]

R: Gene expression data for CD3 cells in blood

|  |  |
| --- | --- |
| CELL {deconf} | R Documentation |

## Gene expression data for CD3 cells in blood

### Description

Gene expression data for 1000 randomly selected genes in CD3 cells (PBMC, blood).
Data are normalized and given in log2-scale. Use together with TISS data and for
the example in function "deconfounding".

### Usage

```
data(CELL)
```

### Format

A data frame with 1000 observations on the following 84 variables.

`c.6.05113820597999..5.90021931507584..7.27941031433168..7.7857002754998..`
:   a numeric vector

`c.6.02303662637988..5.90392612168002..7.42518313780566..8.5938799886972..`
:   a numeric vector

`c.6.00270855005833..5.91184634899326..7.16422647908286..7.46255795372303..`
:   a numeric vector

`c.5.89921656182934..5.82452921825921..7.05160648092607..7.13881099638785..`
:   a numeric vector

`c.5.90103200649364..5.87189244799486..7.27279745773086..7.88728588945808..`
:   a numeric vector

`c.5.93627520781808..5.68514966315495..7.45380524078986..8.07212592000605..`
:   a numeric vector

`c.5.98763879137929..5.95789267059474..7.22512507137255..8.33513006530115..`
:   a numeric vector

`c.6.02883102999355..5.80413314865036..7.42734821138594..8.14130648483892..`
:   a numeric vector

`c.8.60347272691402..8.07443049524315..8.48095529312754..8.73500930097993..`
:   a numeric vector

`c.5.77392858142782..5.66506410067788..7.58089281056473..8.0055636764015..`
:   a numeric vector

`c.6.15236667160367..5.93192740660709..7.35719102823871..8.27585083903127..`
:   a numeric vector

`c.6.00545248159932..5.85622701742989..7.01443151877964..7.77726290545307..`
:   a numeric vector

`c.5.90880839954479..5.82917339688457..7.48715292789157..7.86828969763367..`
:   a numeric vector

`c.5.98196909045981..5.83165962346118..7.1377056515012..7.97007836854138..`
:   a numeric vector

`c.6.03632146226154..5.87352243889388..7.03034754131385..7.48815213614403..`
:   a numeric vector

`c.6.04681112133952..5.97242990973569..7.15604946033465..8.18452757823898..`
:   a numeric vector

`c.5.93660835534094..5.81251898109058..7.85504537045508..7.66695235743765..`
:   a numeric vector

`c.5.95756599088840..5.89727560142517..7.3117558259353..8.13738234370144..`
:   a numeric vector

`c.6.05834686383641..5.8292531538946..6.9737376155394..7.94628480889304..`
:   a numeric vector

`c.6.20377569172702..6.04544782678171..7.05733344297016..7.76631349252909..`
:   a numeric vector

`c.5.98967309482107..5.87106022076027..7.07536424962848..8.15806467834015..`
:   a numeric vector

`c.6.07462743527918..5.99477024160173..7.12750140088176..8.25857629501757..`
:   a numeric vector

`c.5.98597228791227..5.76662147363767..7.67631050800578..8.30160046182044..`
:   a numeric vector

`c.6.19244745307064..5.95974438396367..7.69718837543105..8.96887136427888..`
:   a numeric vector

`c.5.86127494094245..5.78336930692093..7.34091559225257..7.96188825583674..`
:   a numeric vector

`c.6.52527107768049..6.36879102977569..7.69390919962147..8.58767384760791..`
:   a numeric vector

`c.6.01580398776469..5.83848645722721..7.522230696633..8.34247408257067..`
:   a numeric vector

`c.6.08495188161591..5.93130421315264..7.61656506856066..8.07242352349474..`
:   a numeric vector

`c.6.10642073743002..5.91338375074267..7.22686748138091..7.8185513700258..`
:   a numeric vector

`c.6.14890468152762..5.94539657291182..7.27256350295203..8.09008615287528..`
:   a numeric vector

`c.6.0042582668004..5.96227463053816..7.1429563213336..8.03171833441253..`
:   a numeric vector

`c.6.38089536594989..6.17246039847611..7.37141392556655..8.35648866229423..`
:   a numeric vector

`c.6.14228251757454..5.81410852657435..6.95958569914273..7.71395959859612..`
:   a numeric vector

`c.6.19261033145013..6.03513087880445..7.12332099719308..7.56378698423638..`
:   a numeric vector

`c.6.01477145847145..5.88584151387103..7.33004472333418..8.38227338113596..`
:   a numeric vector

`c.6.18621918799101..6.09213392404922..7.47480317644049..8.17988166229393..`
:   a numeric vector

`c.6.06775890695369..5.943553781388..7.32884881950189..8.55550981546475..`
:   a numeric vector

`c.5.90955663551842..5.82967202149800..7.22312311307816..8.20028148787176..`
:   a numeric vector

`c.5.94435689191732..5.88032049014421..7.17778909271036..7.8805237606065..`
:   a numeric vector

`c.5.897805966001..5.91960682383376..6.90699756363868..7.49005796722049..`
:   a numeric vector

`c.5.97645600049408..5.94281040766907..7.32178126485995..8.47142980289147..`
:   a numeric vector

`c.5.67663215418673..5.74965340838778..6.93040751971917..7.21707961639384..`
:   a numeric vector

`c.6.04911833441778..5.9727667108934..6.85992595026922..7.22700883150018..`
:   a numeric vector

`c.5.92335556602201..5.8726239777794..7.15966400093618..7.97833360199343..`
:   a numeric vector

`c.6.2252459667913..6.14253697141312..7.36152531344793..8.63438153337066..`
:   a numeric vector

`c.5.96019118711206..5.79758517687825..7.19400302947994..8.05322153476149..`
:   a numeric vector

`c.6.1432974756548..6.01908510020193..7.06909119425434..8.49944854227708..`
:   a numeric vector

`c.6.07229066999315..5.70535907804591..7.28995260423579..8.01629754034467..`
:   a numeric vector

`c.6.16518671631744..5.94518882366636..7.29778646266848..8.33245487390644..`
:   a numeric vector

`c.5.98983885348554..5.86119080182486..7.03764664395857..7.99003044912555..`
:   a numeric vector

`c.5.79233517489061..5.70189046679349..7.09106625439134..7.62686941014898..`
:   a numeric vector

`c.5.93117921589599..5.7536907192673..6.8070289113143..7.61943147006471..`
:   a numeric vector

`c.6.0545638254291..6.01474631675577..6.9896697949259..7.67290297456182..`
:   a numeric vector

`c.6.06922391187187..5.96399067686076..7.01533486738993..8.13769196098116..`
:   a numeric vector

`c.6.21332145775573..6.04572851006092..7.22208752132478..7.87788737447944..`
:   a numeric vector

`c.5.86278882059521..5.79459486647759..7.19546449006554..8.08415527557463..`
:   a numeric vector

`c.5.99587757179825..5.93231858690848..7.29647607996078..8.20927289665512..`
:   a numeric vector

`c.5.80923611561076..5.802152281186..6.88540207377615..7.78554703971522..`
:   a numeric vector

`c.5.95252135153832..5.87782778624743..7.20999623579468..8.06958465468273..`
:   a numeric vector

`c.6.07316722622771..5.99246265143998..7.31437113578802..7.91968727929064..`
:   a numeric vector

`c.5.99767394694454..5.90079902150872..7.13743086155956..8.2726385533719..`
:   a numeric vector

`c.6.17157124818804..6.03697426063004..7.31032004948047..8.01836304485341..`
:   a numeric vector

`c.5.92227408594866..5.8727147799997..7.42694263737703..8.2130932970732..`
:   a numeric vector

`c.6.01313112339751..5.83165962346118..7.46512019040318..7.92319758957111..`
:   a numeric vector

`c.5.9878042942035..5.9896989397844..6.71721352777491..7.42541671729857..`
:   a numeric vector

`c.5.97430769349514..5.97304538003589..6.8491775931501..7.68348681808..`
:   a numeric vector

`c.5.98214901881223..5.87683984921282..7.43575880473317..8.23575191059264..`
:   a numeric vector

`c.5.99563991440755..5.92203319019117..7.39525338679167..7.64154588944045..`
:   a numeric vector

`c.5.75893969508023..5.71250367939746..7.33344412322032..7.73879525800152..`
:   a numeric vector

`c.5.94216078575838..5.84859820841097..7.23811150945355..8.70059577468986..`
:   a numeric vector

`c.6.00986775245421..5.92710068015933..7.22208752132478..8.24440574154296..`
:   a numeric vector

`c.6.1160836792126..6.01637257641042..7.26287509353881..7.91225281786431..`
:   a numeric vector

`c.6.06358325659496..5.85134558331784..7.32659365799698..7.96769281198301..`
:   a numeric vector

`c.6.1105840643729..5.93202105630917..7.29868597042204..8.21380773399285..`
:   a numeric vector

`c.5.85161650627289..5.81910270105024..6.39404922940972..7.25412989544309..`
:   a numeric vector

`c.6.12788784655999..5.9261848764598..6.4189563808043..6.89097775753145..`
:   a numeric vector

`c.6.01272308510288..5.91634878158057..7.14632602743304..8.13070394590353..`
:   a numeric vector

`c.6.02841724553427..6.11381584472838..6.95529044758918..8.02634365661199..`
:   a numeric vector

`c.6.05844377899806..5.98967309482107..6.96138796495833..7.96327709643457..`
:   a numeric vector

`c.5.98567016106881..5.76433159913111..7.25478294277713..7.8045992226809..`
:   a numeric vector

`c.6.06282639317464..5.97751462643147..7.29289601371908..8.45426504867996..`
:   a numeric vector

`c.6.01027597934577..5.94962766679845..7.30268935422974..8.38898639047276..`
:   a numeric vector

`c.6.07046841243969..5.8853430554679..7.44565739983014..8.23835713488369..`
:   a numeric vector

`c.6.00524321747955..5.86356176726023..6.8993785066561..7.69463876537167..`
:   a numeric vector

### Details

The corresponding dataset is TISS, data from whole blood including CD3 cells.

### Examples

```
data(CELL)
## use as described in the examples for function "deconfounding"
```

---

[Package *deconf* version 1.0 Index]
