# Supplementary material for: Biomarker discovery in heterogeneous tissue samples -taking the in-silico deconfounding approach
Source: BMC Bioinformatics. 2010 Jan 14;11:27. doi: 10.1186/1471-2105-11-27 (PMC3098067; doi:10.1186/1471-2105-11-27)
Supplement: Additional file 1 — R-package deconf(Windows) including example data and script. R-package deconf (Windows version) which implements the deconfounding algorithm together with options for normalization, run-time options for the iteration process, and number of cell-type specific gene expression profiles to be estimated. Also, some toy examples and part of the experimental dataset are included together with executable example scripts for demonstration purposes. [file 1471-2105-11-27-S1.ZIP › deconf/html/deconf-package.html]

R: package deconf contains function "deconfounding", implementing the decomposition
of OMICS datasets of heterogeneous tissues in signature and cell type concentration

|  |  |
| --- | --- |
| deconf-package {deconf} | R Documentation |

## package deconf contains function "deconfounding", implementing the decomposition of OMICS datasets of heterogeneous tissues in signature and cell type concentration

### Description

Main function "deconfounding" implements an iterative decomposition as I = S\*C,
where I is the originally measured data matrix (e.g. genes by samples),
S is the signature matrix (genes by cell types) and C the cell type concentration
matrix (cell types by samples) – see Repsilber et al., 2009.

### Details

|  |  |
| --- | --- |
| Package: | deconf |
| Type: | Package |
| Version: | 1.0 |
| Date: | 2009-09-02 |
| License: | GPL version 2 or later |
| LazyLoad: | yes |

Check help for main function "deconfounding" and its examples.

### Author(s)

Dirk Repsilber <repsilber@fbn-dummerstorf.de>

### References

Repsilber et al., 2009

### See Also

TISS, CELL, stat

### Examples

```
## our simplest example here!
```

---

[Package *deconf* version 1.0 Index]
