# Supplementary material for: Biomarker discovery in heterogeneous tissue samples -taking the in-silico deconfounding approach
Source: BMC Bioinformatics. 2010 Jan 14;11:27. doi: 10.1186/1471-2105-11-27 (PMC3098067; doi:10.1186/1471-2105-11-27)
Supplement: Additional file 1 — R-package deconf(Windows) including example data and script. R-package deconf (Windows version) which implements the deconfounding algorithm together with options for normalization, run-time options for the iteration process, and number of cell-type specific gene expression profiles to be estimated. Also, some toy examples and part of the experimental dataset are included together with executable example scripts for demonstration purposes. [file 1471-2105-11-27-S1.ZIP › deconf/html/deconfounding.html]

R: Function decomposing an OMICS dataset from mixed tissue

|  |  |
| --- | --- |
| deconfounding {deconf} | R Documentation |

## Function decomposing an OMICS dataset from mixed tissue

### Description

The decomposition is according to I = S %\*% C, where I is
the originally measured data matrix, e.g. gene expression matrix,
S is the signature matrix containing as many columns as cell types
and rows as genes, C is the cell type concentration matrix with
as many rows as cell types, and as many columns as samples in the
originally measured dataset.

### Usage

```
deconfounding(I, n.cell.types, n.iterations = 1000, error.threshold = 0)
```

### Arguments

|  |  |
| --- | --- |
| `I` | originally measured data matrix, genes by samples, e.g. gene expression data |
| `n.cell.types` | number of cell types in which the original data should be decomposed |
| `n.iterations` | max number of iterations of the deconfounding algorithm |
| `error.threshold` | min error to continue iterations, if error falls below this threshold, the iterative algorithm stops |

### Details

Data in the originally measured data matrix, I, need to be non-negative.
Try if deconfounding works better in original or log-scale.

### Value

Function deconfounding returns a list of results:

|  |  |
| --- | --- |
| `S` | $Matrix: estimated signature matrix, S |
| `C` | $Matrix: estimated cell type concentration matrix, C |
| `nsim` | number of iterations until the algorithm stopped |
| `error` | error if comparing originally measured data matrix and decomposition S%\*%C |

### Author(s)

Dirk Repsilber, repsilber@fbn-dummerstorf.de

### References

Repsilber et al., 2009

### See Also

TISS, CELL, stat

### Examples

```
library(deconf)
####################################################################
## (1) toy data example:

## cell type proportions matrix:
ct1 <- runif(20)
ct2 <- 1-ct1
CP <- matrix(c(ct1,ct2),ncol=20,nrow=2,byrow=TRUE)

## cell type-specific gene expression matrix:
S.raw <- matrix(rexp(200),ncol=2)

## normalized S matrix:
S <- apply.constraints.S(S.raw)$Matrix

## gene expression matrix (simulated measured intensities):
I <- S 

## deconfound I, using n=2 cell types:
res <- deconfounding(I, n.cell.types=2, n.iterations = 200, error.threshold = 0)

## deconfounding pure random data:
res.rand <- deconfounding(matrix(rexp(100*20),ncol=20), n.cell.types=2, n.iterations = 200, error.threshold = 0)

## display results:
##par(mfrow=c(2,2))
##plot(res$S$Matrix[,1],S[,1],main="S[,1] for cell type 1")
##plot(res$S$Matrix[,1],S[,2],main="S[,2] for cell type 1")
##plot(res$C$Matrix[1,],CP[1,],main="C[1,] for cell type 1")
##plot(res$C$Matrix[1,],CP[2,],main="C[2,] for cell type 1")

####################################################################
## (2) experimental data example:
data(CELL)
data(TISS)
data(stat)
## TISS: expression profiles for 1000 randomly chosen genes, tissue
## CELL: expression profiles for 1000 randomly chosen genes, single cell type
## stat: phenotype data (groups of clinical field study: TB, TSTpos or TSTneg)
## see Repsilber et al., 2009

## select data for TB patients:
I <- TISS[,stat=="TB"]

## run deconfounding:
set.seed(1)
res <- deconfounding(I, n.cell.types=2, n.iterations = 1000, error.threshold = 0)

## check against experimental CD3 profiles in TB patients:
cd3.exp <- CELL[,stat=="TB"]
cd3.exp.mean <- apply(cd3.exp,1,mean)

## plot comparisons:
##par(mfrow=c(1,2))
##plot(cd3.exp.mean,res$S$Matrix[,1],xlim=c(5,16),ylim=c(0,3))
##lines(lowess(x=cd3.exp.mean,y=res$S$Matrix[,1]),col="red")
##abline(lm(res$S$Matrix[,1] ~ cd3.exp.mean),col="green")
##plot(cd3.exp.mean,res$S$Matrix[,2],xlim=c(5,16),ylim=c(0,3))
##lines(lowess(x=cd3.exp.mean,y=res$S$Matrix[,2]),col="red")
##abline(lm(res$S$Matrix[,2] ~ cd3.exp.mean),col="green")
```

---

[Package *deconf* version 1.0 Index]
