# Supplementary material for: Biomarker discovery in heterogeneous tissue samples -taking the in-silico deconfounding approach
Source: BMC Bioinformatics. 2010 Jan 14;11:27. doi: 10.1186/1471-2105-11-27 (PMC3098067; doi:10.1186/1471-2105-11-27)
Supplement: Additional file 1 — R-package deconf(Windows) including example data and script. R-package deconf (Windows version) which implements the deconfounding algorithm together with options for normalization, run-time options for the iteration process, and number of cell-type specific gene expression profiles to be estimated. Also, some toy examples and part of the experimental dataset are included together with executable example scripts for demonstration purposes. [file 1471-2105-11-27-S1.ZIP › deconf/html/stat.html]

R: phenotype data for TISS and CELL datasets

|  |  |
| --- | --- |
| stat {deconf} | R Documentation |

## phenotype data for TISS and CELL datasets

### Description

taking values TB, TSTpos or TSTneg according to three groups
of a clinical field study as examples data for function
"deconfounding" (see examples there)

### Usage

```
data(stat)
```

### Format

The format is:
chr [1:84] "TSTneg" "TSTpos" "TB" "TB" "TSTpos" "TB" ...

### Details

use together with datasets TISS and CELL – see examples of function "deconfounding"

### References

Repsilber et al., 2009

### Examples

```
data(stat)
## see examples for function deconfounding!
```

---

[Package *deconf* version 1.0 Index]
