# Supplementary material for: Biomarker discovery in heterogeneous tissue samples -taking the in-silico deconfounding approach
Source: BMC Bioinformatics. 2010 Jan 14;11:27. doi: 10.1186/1471-2105-11-27 (PMC3098067; doi:10.1186/1471-2105-11-27)
Supplement: Additional file 1 — R-package deconf(Windows) including example data and script. R-package deconf (Windows version) which implements the deconfounding algorithm together with options for normalization, run-time options for the iteration process, and number of cell-type specific gene expression profiles to be estimated. Also, some toy examples and part of the experimental dataset are included together with executable example scripts for demonstration purposes. [file 1471-2105-11-27-S1.ZIP › deconf/html/TISS.html]

R: Example of experimental gene expression data

|  |  |
| --- | --- |
| TISS {deconf} | R Documentation |

## Example of experimental gene expression data

### Description

A data frame with 1000 randomly selected genes from the GC6 dataset described
in Repsilber et al., 2009, article. TISS are gene expression data (log-scale, normalized)
from blood (tissue, mixture of several cell types). Usage as example, see examples for function
deconfounding of this package (deconf).

### Usage

```
data(TISS)
```

### Format

A data frame with 1000 observations on the following 84 variables.

`c.6.08971341383347..6.00471872751374..6.9649755175942..7.07137381928837..`
:   a numeric vector

`c.6.19587844180033..6.0865535489687..7.16830723389662..7.32291043931485..`
:   a numeric vector

`c.6.08194245072898..6.00142312752475..6.72024313637251..7.11130184542762..`
:   a numeric vector

`c.6.20395235084593..5.9559040474712..6.60034401777876..6.83141109504775..`
:   a numeric vector

`c.5.93640935369473..5.81607078959231..6.61616265617036..6.55838338859037..`
:   a numeric vector

`c.6.49599769950502..6.05901100932282..6.83762312055345..6.71572534679945..`
:   a numeric vector

`c.6.47802778638327..6.56207536773..7.1552268852766..7.06279339456836..`
:   a numeric vector

`c.5.9212761257517..5.68880182911026..6.80756866031433..6.67923406365178..`
:   a numeric vector

`c.6.01906266068559..5.86482019334601..6.88470199337524..6.51266170267596..`
:   a numeric vector

`c.6.70938074405827..6.0507195736432..7.0525996702092..7.14241071583926..`
:   a numeric vector

`c.7.01601738260928..6.42696350607342..7.43013346029398..7.4541318592026..`
:   a numeric vector

`c.6.27661956267968..5.95619928267948..7.05216561401357..6.55335388191449..`
:   a numeric vector

`c.7.06793684527728..6.35573691939825..7.41229978850372..7.14918547540999..`
:   a numeric vector

`c.6.17435405893588..5.8770388526654..6.76843059224084..6.56707964719127..`
:   a numeric vector

`c.6.36465507969867..5.8129746517572..6.99020238714289..7.04078795900078..`
:   a numeric vector

`c.6.29834299954479..5.97391795691417..6.80819694066405..6.92869580876474..`
:   a numeric vector

`c.6.44253895602985..5.87581208964519..6.7504190448268..6.80263392885144..`
:   a numeric vector

`c.6.04752595462858..5.88319797632786..6.99760878157354..6.8641826454622..`
:   a numeric vector

`c.6.44167316003576..5.93716123237232..6.7703873665134..6.69829275819179..`
:   a numeric vector

`c.6.49162236885461..5.97396549594117..6.88888932259913..6.7504190448268..`
:   a numeric vector

`c.6.1999089096102..5.72014246506195..6.95565337474523..7.19134925118906..`
:   a numeric vector

`c.6.44364740194515..6.00463752457128..6.99402961396205..7.07320657588836..`
:   a numeric vector

`c.10.2175318910279..7.07193638626913..9.4169496375662..9.46915247308413..`
:   a numeric vector

`c.10.671468121298..7.26976024700268..9.80833159233321..9.9335796174985..`
:   a numeric vector

`c.6.27212199930359..5.90802432266478..7.2204247331196..7.41310182630434..`
:   a numeric vector

`c.6.71128747682889..6.1159218868216..7.30206709471326..7.26597018998593..`
:   a numeric vector

`c.6.30412745312798..5.93338284952987..7.01314431146107..6.95462801631201..`
:   a numeric vector

`c.6.5011115071906..5.92279706319727..6.94597898660284..6.8999047286694..`
:   a numeric vector

`c.6.35218565354121..6.00416988339391..7.1321566102059..7.0128131528178..`
:   a numeric vector

`c.6.15115813499663..6.13385201242930..7.20367585901061..7.35773795503149..`
:   a numeric vector

`c.6.1704318544458..5.79954452761811..6.9818186804304..7.05314027864823..`
:   a numeric vector

`c.5.96595301094727..5.75796320727096..6.52960040969749..6.65738263542997..`
:   a numeric vector

`c.6.18737568827696..5.8597896993449..6.97973460599958..6.88703139014556..`
:   a numeric vector

`c.6.16172990605101..5.86202746363999..6.8762805226837..6.89031955403056..`
:   a numeric vector

`c.6.18870859742927..5.68406232274515..6.83307154479276..6.81210674279434..`
:   a numeric vector

`c.6.30867485874417..5.88660376200534..6.63529421262821..7.00238851490023..`
:   a numeric vector

`c.6.06454165387574..5.83900162955304..6.88480217564964..6.98687222312216..`
:   a numeric vector

`c.6.12721174431123..5.82068520635617..6.86075170340456..7.00597260199497..`
:   a numeric vector

`c.6.14957434373828..5.80699546278887..6.64986981516468..6.93423935417161..`
:   a numeric vector

`c.6.16880151134689..5.79503401997352..7.05804841776274..7.38261920125272..`
:   a numeric vector

`c.6.08280267334169..5.90615548916635..6.79697082366922..7.10613361957809..`
:   a numeric vector

`c.6.13530004762873..5.83999440247044..7.08808021660078..7.07205738447418..`
:   a numeric vector

`c.6.26806000669119..6.04997919553546..7.1288044096574..7.04630774286057..`
:   a numeric vector

`c.6.14625259530153..5.76329425373752..6.77172757315479..6.81669077176976..`
:   a numeric vector

`c.6.17884664400478..6.06192825107784..6.98580484015131..7.09456338826518..`
:   a numeric vector

`c.6.18998656689162..5.84549688401033..6.71954140184481..7.13160843578875..`
:   a numeric vector

`c.6.03669788566798..5.94647950409069..6.75919632958298..7.25892030051775..`
:   a numeric vector

`c.6.15743473394172..5.97074431093009..6.84211197155421..6.83509883855999..`
:   a numeric vector

`c.6.45144385344929..6.60264414443102..7.01707700626918..7.17255673738775..`
:   a numeric vector

`c.6.39857044406542..5.95445507407645..6.68967183191815..6.79620880861387..`
:   a numeric vector

`c.7.34162216546292..6.41031690363396..7.23933943226399..7.30849391494733..`
:   a numeric vector

`c.6.19364056422936..5.77512877054322..6.89440211016725..7.15450290479949..`
:   a numeric vector

`c.6.31076114963664..6.03481600837812..6.8301097659786..7.15439381509907..`
:   a numeric vector

`c.6.16130924415785..6.02325088897474..6.92070478824317..6.83785492446582..`
:   a numeric vector

`c.6.34722052597755..5.96293725397923..6.86428683048232..6.86740913463034..`
:   a numeric vector

`c.6.24972103635164..5.83124816548947..7.06498105179445..7.2722573742958..`
:   a numeric vector

`c.6.02711262005754..5.86172162786226..6.85484355259333..7.02754830095897..`
:   a numeric vector

`c.5.95154141214294..5.89906944785221..6.5307998901576..6.80409331782419..`
:   a numeric vector

`c.5.97927450366971..6.00578579488318..6.56608853456432..7.17102670621887..`
:   a numeric vector

`c.6.38108308810618..6.13397550624798..7.09737677455225..7.57195409836484..`
:   a numeric vector

`c.6.03948092993013..5.94175182212763..6.70271106285088..7.25734334711813..`
:   a numeric vector

`c.6.2021599252215..6.07240077299518..6.69211566747936..7.04598284425192..`
:   a numeric vector

`c.6.14694088377147..5.76443082540343..6.55859346917383..6.79027552304646..`
:   a numeric vector

`c.6.22974357804233..5.9683298530456..6.97496757024913..7.033405546061..`
:   a numeric vector

`c.6.09759972155112..5.85167736902203..6.67914522571332..6.9236253510978..`
:   a numeric vector

`c.6.16017326437743..6.08271737742829..6.91143785153476..7.24523494744328..`
:   a numeric vector

`c.6.16188747355579..5.91872781449377..6.65435610559622..6.613578536498..`
:   a numeric vector

`c.6.82621790860338..6.48342106935302..7.09044153952881..7.22803439689266..`
:   a numeric vector

`c.6.27737161330805..5.91628018457662..6.80468232606215..7.0500085993882..`
:   a numeric vector

`c.6.4881010279327..6.03921365798576..7.05197070488834..7.62368780778255..`
:   a numeric vector

`c.6.26407247905406..5.87902457918331..6.92879678813109..7.06137114008675..`
:   a numeric vector

`c.6.35180669836784..5.93904643971666..6.84380600131104..7.05411853241592..`
:   a numeric vector

`c.6.1317645127849..5.896817379933..7.1499895692605..7.39097053760625..`
:   a numeric vector

`c.6.45886379211413..6.21278250967252..7.39182695605004..7.58646735338602..`
:   a numeric vector

`c.6.14116041715101..5.75902184642643..7.3567783076516..7.89984494700946..`
:   a numeric vector

`c.6.31295526783908..6.03571726360607..7.17465936001145..7.28642891493815..`
:   a numeric vector

`c.6.40683197158285..6.10050901010969..7.1633125043997..7.46059936525042..`
:   a numeric vector

`c.6.33086337113321..6.07974613485608..7.1526701405761..7.50352964158517..`
:   a numeric vector

`c.6.52211441909484..5.87614925208669..6.94367159646729..6.91063409024364..`
:   a numeric vector

`c.6.21012900759257..5.91099913250998..7.13801106469537..7.54357286749005..`
:   a numeric vector

`c.6.19591574905616..5.99666991824106..6.81937073725626..7.04758675311804..`
:   a numeric vector

`c.6.3530542998948..6.10529125415735..6.95523442621864..7.30849391494733..`
:   a numeric vector

`c.6.0002414505678..5.95133929844098..6.97028517560065..7.37059342415298..`
:   a numeric vector

`c.6.15203860787798..6.03138890861212..6.915781547884..7.05973634509311..`
:   a numeric vector

### Details

The corresponding dataset for validation of the deconfounding results is CELL (this package).

### Source

Repsilber et al., 2009

### Examples

```
data(TISS)
## use together with function "deconfounding", see examples there.
```

---

[Package *deconf* version 1.0 Index]
